# Supplementary material for: Modeling glioblastoma heterogeneity as a dynamic network of cell states
Source: Mol Syst Biol. 2021 Sep 16;17(9):e10105. doi: 10.15252/msb.202010105 (PMC8444284; doi:10.15252/msb.202010105)
Supplement: Supplementary file 6 — Source Data for Figure 5 [file MSB-17-e10105-s004.zip › Figure5A_sourcedata/GSEA_3017/nosigdb_stateA.GseaPreranked.1622795026485/FELDMAN_2020_G2-M.html]

Details for gene set FELDMAN\_2020\_G2-M[GSEA]

|  || Dataset | state53017 |
| Phenotype | NoPhenotypeAvailable |
| Upregulated in class | na\_pos |
| GeneSet | FELDMAN\_2020\_G2-M |
| Enrichment Score (ES) | 0.32551655 |
| Normalized Enrichment Score (NES) | 2.5735152 |
| Nominal p-value | 0.0 |
| FDR q-value | 5.5023927E-5 |
| FWER p-Value | 0.001 |
Table: GSEA Results Summary

  

Fig 1: Enrichment plot: FELDMAN\_2020\_G2-M      
 Profile of the Running ES Score & Positions of GeneSet Members on the Rank Ordered List

  

| PROBE | GENE SYMBOL | GENE\_TITLE | RANK IN GENE LIST | RANK METRIC SCORE | RUNNING ES | CORE ENRICHMENT || 1 | CDK1 |  |  | 3 | 1.006 | 0.0133 | Yes |
| 2 | MKI67 |  |  | 4 | 0.926 | 0.0287 | Yes |
| 3 | KIF11 |  |  | 6 | 0.870 | 0.0421 | Yes |
| 4 | UBE2C |  |  | 7 | 0.793 | 0.0553 | Yes |
| 5 | KIF20B |  |  | 10 | 0.749 | 0.0655 | Yes |
| 6 | CEP55 |  |  | 11 | 0.747 | 0.0779 | Yes |
| 7 | BUB3 |  |  | 12 | 0.740 | 0.0903 | Yes |
| 8 | KIF5B |  |  | 16 | 0.687 | 0.0982 | Yes |
| 9 | KPNA2 |  |  | 20 | 0.667 | 0.1059 | Yes |
| 10 | IGFBP3 |  |  | 26 | 0.641 | 0.1107 | Yes |
| 11 | ASPM |  |  | 27 | 0.639 | 0.1214 | Yes |
| 12 | PIF1 |  |  | 30 | 0.627 | 0.1295 | Yes |
| 13 | CCNB1 |  |  | 37 | 0.599 | 0.1325 | Yes |
| 14 | CKS2 |  |  | 40 | 0.587 | 0.1399 | Yes |
| 15 | HMMR |  |  | 43 | 0.559 | 0.1469 | Yes |
| 16 | NCAPG |  |  | 44 | 0.556 | 0.1562 | Yes |
| 17 | HJURP |  |  | 47 | 0.539 | 0.1628 | Yes |
| 18 | CKS1B |  |  | 50 | 0.530 | 0.1693 | Yes |
| 19 | DBF4 |  |  | 55 | 0.514 | 0.1732 | Yes |
| 20 | KIF14 |  |  | 59 | 0.503 | 0.1781 | Yes |
| 21 | CENPE |  |  | 62 | 0.496 | 0.1841 | Yes |
| 22 | MIS18BP1 |  |  | 64 | 0.495 | 0.1911 | Yes |
| 23 | CCNA2 |  |  | 65 | 0.495 | 0.1994 | Yes |
| 24 | TPX2 |  |  | 66 | 0.488 | 0.2075 | Yes |
| 25 | FGFBP3 |  |  | 75 | 0.477 | 0.2061 | Yes |
| 26 | DLGAP5 |  |  | 76 | 0.475 | 0.2140 | Yes |
| 27 | AURKA |  |  | 78 | 0.474 | 0.2208 | Yes |
| 28 | CDC20 |  |  | 80 | 0.472 | 0.2275 | Yes |
| 29 | UBE2T |  |  | 83 | 0.470 | 0.2330 | Yes |
| 30 | PLK1 |  |  | 89 | 0.464 | 0.2349 | Yes |
| 31 | TUBB4B |  |  | 93 | 0.462 | 0.2391 | Yes |
| 32 | CENPF |  |  | 94 | 0.461 | 0.2468 | Yes |
| 33 | TOP2A |  |  | 95 | 0.457 | 0.2544 | Yes |
| 34 | ZWINT |  |  | 106 | 0.447 | 0.2501 | Yes |
| 35 | AURKB |  |  | 108 | 0.446 | 0.2564 | Yes |
| 36 | CDCA8 |  |  | 115 | 0.437 | 0.2567 | Yes |
| 37 | KIF23 |  |  | 116 | 0.436 | 0.2640 | Yes |
| 38 | DEPDC1 |  |  | 119 | 0.432 | 0.2688 | Yes |
| 39 | MELK |  |  | 123 | 0.427 | 0.2724 | Yes |
| 40 | BUB1B |  |  | 129 | 0.421 | 0.2736 | Yes |
| 41 | RTKN2 |  |  | 141 | 0.413 | 0.2676 | Yes |
| 42 | GTSE1 |  |  | 146 | 0.411 | 0.2698 | Yes |
| 43 | CCNB2 |  |  | 147 | 0.411 | 0.2767 | Yes |
| 44 | ANLN |  |  | 152 | 0.407 | 0.2788 | Yes |
| 45 | MAD2L1 |  |  | 153 | 0.406 | 0.2855 | Yes |
| 46 | TNC |  |  | 159 | 0.402 | 0.2864 | Yes |
| 47 | KIFC1 |  |  | 160 | 0.402 | 0.2931 | Yes |
| 48 | ARHGAP11A |  |  | 162 | 0.399 | 0.2986 | Yes |
| 49 | KIF2C |  |  | 165 | 0.397 | 0.3029 | Yes |
| 50 | NDC80 |  |  | 173 | 0.393 | 0.3012 | Yes |
| 51 | FAM83D |  |  | 180 | 0.389 | 0.3007 | Yes |
| 52 | HNRNPH3 |  |  | 185 | 0.387 | 0.3025 | Yes |
| 53 | METRN |  |  | 186 | 0.387 | 0.3089 | Yes |
| 54 | BUB1 |  |  | 188 | 0.384 | 0.3142 | Yes |
| 55 | KIF22 |  |  | 197 | 0.377 | 0.3111 | Yes |
| 56 | CDCA3 |  |  | 203 | 0.372 | 0.3115 | Yes |
| 57 | PTMA |  |  | 206 | 0.371 | 0.3153 | Yes |
| 58 | TRIP13 |  |  | 209 | 0.370 | 0.3192 | Yes |
| 59 | SMC4 |  |  | 217 | 0.367 | 0.3171 | Yes |
| 60 | RAB13 |  |  | 220 | 0.366 | 0.3208 | Yes |
| 61 | SPC25 |  |  | 226 | 0.363 | 0.3211 | Yes |
| 62 | CENPA |  |  | 243 | 0.352 | 0.3082 | Yes |
| 63 | H2AFZ |  |  | 245 | 0.351 | 0.3129 | Yes |
| 64 | TTK |  |  | 262 | 0.340 | 0.2998 | Yes |
| 65 | KNSTRN |  |  | 263 | 0.340 | 0.3055 | Yes |
| 66 | TUBB |  |  | 264 | 0.340 | 0.3112 | Yes |
| 67 | SLC25A5 |  |  | 266 | 0.340 | 0.3157 | Yes |
| 68 | PLP1 |  |  | 272 | 0.338 | 0.3155 | Yes |
| 69 | LRR1 |  |  | 273 | 0.337 | 0.3211 | Yes |
| 70 | DEPDC1B |  |  | 275 | 0.335 | 0.3255 | Yes |
| 71 | ESCO2 |  |  | 282 | 0.333 | 0.3240 | No |
| 72 | CACYBP |  |  | 288 | 0.330 | 0.3237 | No |
| 73 | G2E3 |  |  | 302 | 0.325 | 0.3139 | No |
| 74 | CENPN |  |  | 310 | 0.323 | 0.3111 | No |
| 75 | HNRNPDL |  |  | 315 | 0.320 | 0.3117 | No |
| 76 | TNFAIP8L1 |  |  | 336 | 0.314 | 0.2936 | No |
| 77 | CLIC1 |  |  | 342 | 0.311 | 0.2929 | No |
| 78 | PRC1 |  |  | 350 | 0.310 | 0.2899 | No |
| 79 | ADA |  |  | 353 | 0.308 | 0.2927 | No |
| 80 | ANP32B |  |  | 357 | 0.307 | 0.2943 | No |
| 81 | ECT2 |  |  | 365 | 0.305 | 0.2912 | No |
| 82 | CDCA2 |  |  | 369 | 0.304 | 0.2927 | No |
| 83 | CKAP2 |  |  | 379 | 0.301 | 0.2872 | No |
| 84 | RPA3 |  |  | 381 | 0.300 | 0.2911 | No |
| 85 | RRM2 |  |  | 383 | 0.300 | 0.2949 | No |
| 86 | CENPW |  |  | 386 | 0.299 | 0.2975 | No |
| 87 | PHF19 |  |  | 393 | 0.297 | 0.2955 | No |
| 88 | HMGA1 |  |  | 397 | 0.295 | 0.2969 | No |
| 89 | RACGAP1 |  |  | 399 | 0.293 | 0.3006 | No |
| 90 | HMGB1 |  |  | 403 | 0.292 | 0.3019 | No |
| 91 | CCT5 |  |  | 409 | 0.290 | 0.3009 | No |
| 92 | DCP2 |  |  | 416 | 0.289 | 0.2987 | No |
| 93 | HMGB2 |  |  | 424 | 0.288 | 0.2953 | No |
| 94 | NUSAP1 |  |  | 430 | 0.286 | 0.2943 | No |
| 95 | NUF2 |  |  | 434 | 0.285 | 0.2955 | No |
| 96 | MIS18A |  |  | 439 | 0.284 | 0.2956 | No |
| 97 | NCAPD2 |  |  | 440 | 0.283 | 0.3003 | No |
| 98 | LMNB1 |  |  | 442 | 0.282 | 0.3038 | No |
| 99 | KIF20A |  |  | 447 | 0.281 | 0.3038 | No |
| 100 | ANP32E |  |  | 453 | 0.280 | 0.3027 | No |
| 101 | MT2A |  |  | 473 | 0.275 | 0.2850 | No |
| 102 | SHCBP1 |  |  | 477 | 0.274 | 0.2860 | No |
| 103 | KIF4A |  |  | 504 | 0.269 | 0.2601 | No |
| 104 | UBE2S |  |  | 507 | 0.269 | 0.2622 | No |
| 105 | SKA3 |  |  | 531 | 0.264 | 0.2397 | No |
| 106 | RANBP1 |  |  | 536 | 0.263 | 0.2394 | No |
| 107 | CDKN3 |  |  | 539 | 0.263 | 0.2415 | No |
| 108 | FBXO5 |  |  | 557 | 0.258 | 0.2259 | No |
| 109 | SPDL1 |  |  | 560 | 0.258 | 0.2278 | No |
| 110 | DDX39A |  |  | 562 | 0.258 | 0.2310 | No |
| 111 | RPL31 |  |  | 568 | 0.256 | 0.2294 | No |
| 112 | TMPO |  |  | 584 | 0.253 | 0.2160 | No |
| 113 | PLIN3 |  |  | 619 | -0.259 | 0.1806 | No |
| 114 | S100B |  |  | 696 | -0.299 | 0.0966 | No |
| 115 | MALAT1 |  |  | 702 | -0.302 | 0.0957 | No |
| 116 | IGFBP7 |  |  | 780 | -0.368 | 0.0117 | No |
| 117 | SPARC |  |  | 782 | -0.369 | 0.0167 | No |
| 118 | DCBLD2 |  |  | 791 | -0.375 | 0.0136 | No |
| 119 | BEX1 |  |  | 802 | -0.385 | 0.0083 | No |
| 120 | FTL |  |  | 825 | -0.409 | -0.0106 | No |
| 121 | FILIP1L |  |  | 835 | -0.428 | -0.0140 | No |
| 122 | CALD1 |  |  | 889 | -0.527 | -0.0673 | No |
| 123 | GPM6B |  |  | 897 | -0.542 | -0.0665 | No |
| 124 | SAT1 |  |  | 909 | -0.573 | -0.0698 | No |
| 125 | FTH1 |  |  | 910 | -0.573 | -0.0602 | No |
| 126 | TTYH1 |  |  | 932 | -0.652 | -0.0739 | No |
| 127 | CST3 |  |  | 933 | -0.680 | -0.0626 | No |
| 128 | CLU |  |  | 938 | -0.685 | -0.0558 | No |
| 129 | SERPINE1 |  |  | 951 | -0.774 | -0.0570 | No |
| 130 | IFITM3 |  |  | 954 | -0.830 | -0.0454 | No |
| 131 | CDKN1A |  |  | 969 | -0.987 | -0.0454 | No |
| 132 | FABP7 |  |  | 971 | -0.994 | -0.0300 | No |
| 133 | CTGF |  |  | 976 | -1.182 | -0.0149 | No |
| 134 | IGFBP5 |  |  | 983 | -1.595 | 0.0047 | No |
Table: GSEA details [plain text format]

  

Fig 2: FELDMAN\_2020\_G2-M: Random ES distribution      
 Gene set null distribution of ES for **FELDMAN\_2020\_G2-M**
